# Involvement of phenoloxidase in browning during grinding of *Tenebrio molitor* larvae

**DOI:** 10.1371/journal.pone.0189685

**Published:** 2017-12-15

**Authors:** Renske H. Janssen, Catriona M. M. Lakemond, Vincenzo Fogliano, Giovanni Renzone, Andrea Scaloni, Jean-Paul Vincken

**Affiliations:** 1 Food Quality and Design, Wageningen University, Wageningen, The Netherlands; 2 Laboratory of Food Chemistry, Wageningen University, Wageningen, The Netherlands; 3 Proteomics & Mass Spectrometry Laboratory, ISPAAM, National Research Council, Naples, Italy; Pusan National University, REPUBLIC OF KOREA

## Abstract

Insects are investigated as alternative protein source to meet the increasing demand for proteins in the future. Enzymatic browning occurring during grinding of insect and subsequent extraction of proteins can influence the proteins’ properties, but it is unclear which enzymes are responsible for this phenomenon. This study was performed on larvae of three commonly used insect species, namely *Tenebrio molitor*, *Alphitobius diaperinus* and *Hermetia illucens*. Oxygen consumption measurements on protein extracts showed activity on L-tyrosine, L-3,4-di-hydroxy-phenylalanine (L-DOPA) and L-dopamine, indicating phenoloxidase as a key player in browning. Furthermore, no reaction on 2,2'-azino-bis(3-ethylbenzothiazoline-6-sulphonic acid) was observed, ruling out an important contribution of laccase to browning. The browning reaction was most prominent at pH 6 for *T*. *molitor* and *A*. *diaperinus*, and 7 for *H*. *illucens*. As the enzyme activity of *H*. *illucens* was the lowest with the darkest color formation, this was likely caused by another factor. The activity of phenoloxidase was confirmed for *T*. *molitor* and *A*. *diaperinus* by activity measurements after fractionation by anion-exchange chromatography. Color measurements showed the presence of activity on both L-DOPA and L-tyrosine in the same fractions. Both substrates were converted into dopachrome after incubation with enzyme-enriched fractions. No DOPA-decarboxylase, tyrosine hydroxylase and peroxidase activities were observed. By using native PAGE with L-DOPA as staining-solution, active *T*. *molitor* protein bands were resolved and characterized, identifying a tyrosinase/phenoloxidase as the active enzyme species. All together, these data confirmed that tyrosinase is an important enzyme in causing enzymatic browning in *T*. *molitor* and likely in *A*. *diaperinus*.

## Introduction

The United Nations has predicted that the world population will reach 9.7 billion people by 2050 [1]. To feed this growing population, new and sustainable protein sources are needed. Insects might be a potential sustainable source of novel proteins, as they contain between 30% and 70% proteins on dry matter basis, and have high quality proteins in terms of amino acid composition [2]. Although insects are consumed in some parts of the world, Westerners dislike them as protein sources. This might change when insects are added as ingredient so that they are not recognizable as such [3]. To use insects as ingredient, often they need to be ground and substantial enzymatic browning occurs [4]. During enzymatic browning phenolic compounds are oxidized, and can further react non-enzymatically into brown pigments or react with proteins and amino acids [5–7]. Browning can influence insect applicability as food ingredient in four different ways.

(i) The visual appearance of browning is often linked to deterioration of foods and will decrease the economic value of food products as is often the case with fruits, vegetables or shrimps [8]. (ii) Browning can also deteriorate the flavor [9]. (iii) Quinones can react with proteins and might decrease their digestibility and its corresponding nutritional quality [9–11]. The presence of melanization-engaging proteins, which can react with quinones were already shown in *T*. *molitor* before [12]. (iv) Browning of proteins can affect their solubility and techno-functional properties, like foaming and emulsifications characteristics [11]. Also protein-chitosan crosslinking might occur and alter techno-functional properties [13].

**Fig 1:**
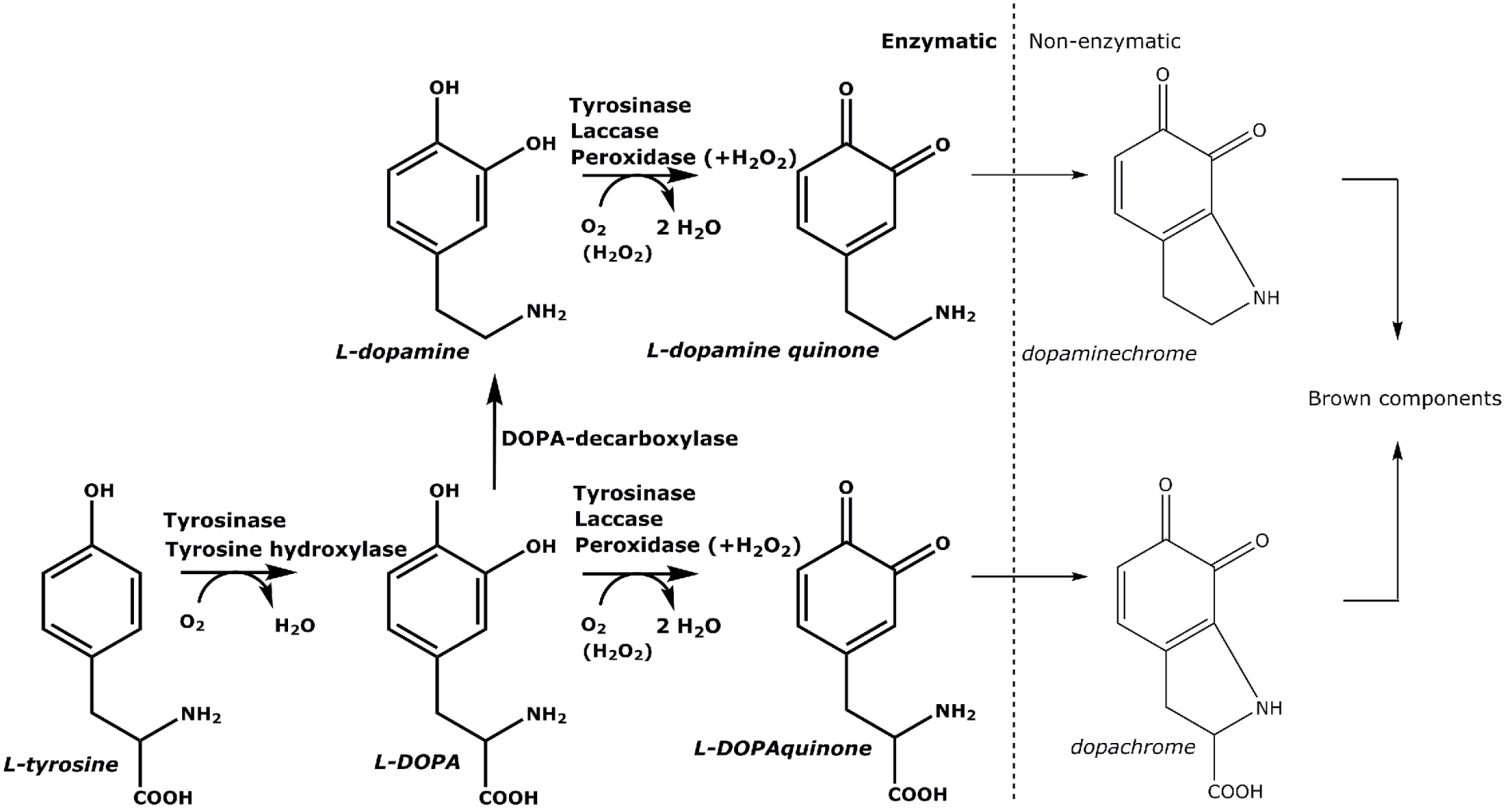
Tyrosine-related substrates, reactions and enzymes involved in enzymatic browning in insects, together with subsequent non-enzymatic reactions.

As shown in Fig 1, five different enzymes can play a role in enzymatic browning in insects, namely phenoloxidase, laccase, tyrosine hydroxylase, DOPA decarboxylase and peroxidase [14,15]. In insects, these enzymes are related to defense mechanisms and can quickly react upon biotic/abiotic stresses, such as bacterial challenges or animal wounding. Besides, these enzymes are known to participate in exoskeletal sclerotization [14,16].

Phenoloxidase, in insects called phenol oxidase, hydroxylates monophenols (e.g., L-tyrosine) to generate corresponding *ortho*-diphenols, and subsequently oxidizes them into *ortho*-quinones [14]. These quinones can further react non-enzymatically with proteins and/or form melanins. The hydroxylation involves a lag-period, which can be shortened by the presence of diphenols [17]. Tyrosinase involvement was demonstrated in sclerotization in *Tribolium castaneum* [18]. Furthermore, conversion of prophenoloxidase into active phenoloxidase was associated with the activation of the immune response in *T*. *molitor* [19]. On the other hand, laccase cannot hydroxylate monophenols, but only oxidize *para*- and *ortho*-diphenols into quinones. This enzyme (or family of enzymes) is linked to the cuticular structure and is responsible for corresponding sclerotization of insects [14]. Tyrosine hydroxylase can only hydroxylate monophenols into diphenols, but does not oxidize diphenols further. It was shown that this enzyme is also involved in sclerotization and brown pigmentation of the insect cuticle in *T*. *castaneum* [20]. DOPA decarboxylase converts L-DOPA into L-dopamine, which is a better substrate for phenoloxidase; this enzyme process was activated as immune response in *T*. *molitor* upon bacterial injection [16,21]. All previously mentioned enzymes use oxygen for substrate modification, whereas peroxidase oxidizes phenols only in the presence of hydrogen peroxide. Peroxidase was also demonstrated to be involved in cuticular sclerotization [14].

In order to assign the main enzymatic activity (or activities) responsible for enzymatic browning during grinding, an activity-guided approach was applied to the larvae of three commonly used insect species. This research focused on the relevant enzymes in the whole larvae that cause above-mentioned phenomena, impairing the use of insect species *T*. *molitor* (yellow mealworm), *A*. *diaperinus* (lesser mealworm) and *H*. *illucens* (black soldier fly) for food and feed applications. Furthermore, no deliberate prior activation from inactive prophenoloxidase into phenoloxidase was performed, in order to reflect the events occurring during grinding of insects for protein extraction as closely as possible.

## Materials and methods

*T*. *molitor* and *A*. *diaperinus* larvae were purchased from Kreca Ento-feed BV (Ermelo, The Netherlands). *H*. *illucens* larvae were kindly provided by the Laboratory of Entomology, (Wageningen University, The Netherlands). Larvae were frozen using liquid nitrogen and stored at -20°C. Water was prepared using a Milli-Q water purification system (Millipore, Billerica, MA, USA). L-tyrosine, sodium phosphate dibasic dihydrate (Na_2_HPO_4_), hydrogen peroxide, tris(hydroxymethyl)aminomethane and glycerol were purchased from Merck (Darmstadt, Germany). Ultra-high-performance liquid chromatography/mass spectrometry (UHPLC/MS) grade formic acid, acetonitrile (ACN) and water were purchased from Biosolve BV (Valkenswaard, The Netherlands). Trypsin was of sequencing grade from Roche (Mannheim, Germany). All solvents for nano-chromatographic analyses were of LC-MS grade from Millipore. All other chemicals used were purchased from Sigma-Aldrich (St. Louis, MO, USA).

### Color formation of insect protein extracts

Frozen larvae were ground in MilliQ water with a kitchen blender (Tomado TM-2419, Oosterhout, NL), using an insect material to solution ratio 1:4 w/v. After centrifugation at 22,000 *g* (5 min, at 4°C), a picture was taken in a photo box.

### Enzyme extraction at different pH values

Enzymes were extracted from insect larvae with 0.1 M citric acid / 0.2 M Na_2_HPO_4_ buffer with pH values in the range of pH 4–7. Frozen larvae were ground in buffer with a kitchen blender (Tomado TM-2419, Oosterhout, NL), using an insect material to solution ratio 1:4 w/v. The mixtures were centrifuged at 22,000 *g* (5 min, at 4°C). This resulted in three layers: pellet, supernatant and fat layer. Supernatants were centrifuged again under similar conditions, and then used to assay enzymatic activities by oxygen consumption measurements.

### Enzyme activity using oxygen consumption measurements

Oxygen consumption was measured with an Oxytherm System (Hansatech, Kings Lynn, UK). The enzyme extract (50 μL) was added to 1 mL of substrate in 0.1 M citric acid / 0.2 M Na_2_HPO_4_ buffer of pH 4–7, at 25°C. The substrates used were 3 mM L-DOPA with or without 0.01% H_2_O_2_, 3 mM L-dopamine, 1 mM L-tyrosine and 3 mM 2,2'-azino-bis(3-ethylbenzothiazoline-6-sulphonic acid) (ABTS). The activity of specific enzymes on different substrates is shown in Table 1. Data acquisition and analysis were done using O_2_ view 2.05 software (Hansatech, King Lynn, UK). The oxygen consumption rate was calculated using the slope of the linear part of the oxygen consumption versus time plot.

**Table 1:** Overview of reactions between different enzymes and substrates which occur (+).

	L-tyrosine	L-DOPA	ABTS	L-DOPA+H_2_O_2_	L-dopamine
Tyrosinase	+	+	-	+	+
Laccase	-	+	+	+	+
Tyrosine hydroxylase	+	-	-	-	-
DOPA decarboxylase	-	+	-	+	-
Peroxidase	-	-	-	+	-

### Enzyme extraction from insects for further fractionation

For enzyme extraction, frozen insect larvae were blended with a kitchen blender (Tomado TM-2419, Oosterhout, NL) in 15 mM phosphate buffer, pH 6, using an insect material to solution ratio 1:4 w/v. These mixtures were centrifuged at 22,000 *g* (5 min, at 4°C). Then, the supernatants were filtered through Whatman filter paper 595 ½ (Schleicher&Schnell, Dassel, Germany) and the filtrates were centrifuged at 22,000 *g* (10 min, at 4°C). These materials constitute the protein extracts used for further enzyme fractionations. It is noted that the enzyme extract of *T*. *molitor* retained almost 80% of its activity for 2 weeks when stored at 4°C. It was therefore not necessary to perform protein activation treatments.

### Enzyme fractionation by anion exchange chromatography

Anion-exchange chromatography (AEC) was used to separate insect proteins. Insect extracts (0.5 mL) were manually injected onto a 1 mL Resource Q column (GE Healthcare, Sweden) on the ÄKTA micro (Amersham Biosciences, UK). The column was equilibrated with 15 mM phosphate buffer, pH 6 or 7. A linear gradient up to 1 M NaCl in the same buffer was applied over 20 column volumes, using a flow rate of 0.5 mL/min. Fractions of 1 mL were collected using a fraction collector (GE FRAC-950, Amersham Biosciences, UK). UV absorbance was measured at 214 nm.

### Enzyme activity measurements using spectrophotometry

Enzyme fractions (50 μL) were incubated with 200 μL of different substrates, namely 3 mM L-DOPA, 1 mM L-tyrosine, 3 mM ABTS and 3 mM L-DOPA with 0.01% H_2_O_2_. A time-course absorbance measurement at 520 nm (or at 420 nm when ABTS was used as substrate) was obtained with a spectrophotometer (Tecan Infinite F500, Tecan, Switzerland).

### Pooling anion-exchange chromatography fractions

Multiple fractions associated with the same enzyme activity were pooled. The fractions T1-3 were pooled in T_I_, T4-8 in T_II_, T9-12 in T_III_, T13-17 in T_IV_ and T18-23 in T_V_. These AEC pools were concentrated using centrifugal filter units (Amicon Ultra, 0.5 mL, 10 kDa molecular mass cut off, Millipore, Ireland) and used for further assays based on UHPLC-MS analysis and native PAGE.

### Reversed phase-ultra high performance liquid chromatography-electrospray ionisation—mass spectrometry (RP-UHPLC-ESI-MS) analysis of enzymatic reactions

Concentrated pools (25 μL) were incubated with 200 μL substrates, *i*.*e*. 0.05 mg/mL L-DOPA or L-tyrosine. Standards of L-DOPA, L-dopamine and L-tyrosine were used for calibration purpose. Furthermore, a standard incubation using L-DOPA and purified mushroom phenoloxidase was used to determine the product dopachrome.

After incubation, samples were analyzed with an Accela UHPLC system (Thermo Scientific, USA), which was equipped with a pump, an auto sampler and a photo-diode array detector (PDA). Each sample (5 μL) was injected onto a Hypersil Gold aQ column (2.1 x 150 mm, particle size 1.9 μm; Thermo Scientific, USA), and eluted with UHPLC-grade 0.1% v/v formic acid (eluent A) and UHPLC-grade acetonitrile, containing 0.1% v/v formic acid (eluent B). The flow rate was 400 μL/min; the temperature of the column oven and of the tray was set at 10°C. The PDA detector was set to measure over the range of 200–600 nm. The following gradient was used: 0–5 min, isocratic on 100% v/v A; 5–19.57 min, linear gradient from 0% to 50% v/v B; 19.57–20.15 min, linear gradient from 50% to 100% v/v B; 20.15–23.06 min, isocratic on 100% v/v B; 23.06–23.64 min, linear gradient from 100% to 0% v/v B; 23.64–28.09, isocratic on 100% v/v A. Mass spectrometric data were obtained by analyzing samples on a LTQ-XL (Thermo Scientific, USA) equipped with an electrospray probe coupled to the UHPLC system. The source voltage was 3.5 kV in the negative ion mode and 4 kV in the positive ion mode. The temperature of the ion transfer tube was 250°C. The instrument was tuned using L-DOPA and L-tyrosine. Data were collected over the *m/z* range 150–300. Tandem mass spectra were collected with a collision energy of 30%. Control of the instrument and analysis of the data were done using Xcalibur 2.2 (Thermo Scientific, USA). Quantitative data were obtained from comparison of enzymatic reactions with calibration curves of specific metabolite standards.

### Native PAGE analysis

Native PAGE was performed under non-reducing conditions with Bio-Rad any kDa, Mini-protean TGX precast protein gel in a Mini-Protean II system (Bio-Rad laboratories), according to the manufacturer’s protocol. Samples were 10 times concentrated by ultrafiltration using centrifugal filters (Amicon Ultra, 0.5 mL, 10 kDa molecular mass cut off, Millipore, Ireland) and mixed 1:1 ratio with sample buffer. For the fractions, 20 μL-samples were loaded onto the gel. For the total extract, a 10 μL-sample was loaded onto the gel. Resulting gels were stained with 3 mM L-DOPA in MilliQ for at least 2.5 h or with Instant Blue (Expedeon, UK).

### Proteomic analysis of native PAGE bands

Visible *T*. *molitor* enzyme bands from native PAGE with L-DOPA staining were carefully cut, *in gel*-reduced with dithiothreitol, *S*-alkylated with iodoacetamide, and subsequently *in-gel* digested with trypsin [22]. Resulting peptide mixtures were desalted with μZip-TipC18 micro-columns (Millipore), and then subjected to nanoLC-ESI-LIT-MS/MS analysis. The latter was performed with a LTQ XL mass spectrometer (Thermo Scientific, USA) equipped with a Proxeon nano-spray source (Thermo Scientific, USA) connected to an UltiMate 3000 RSLC nano-liquid chromatographer (Dionex, Thermo Scientific, USA). Protein digests were resolved on a 15 cm length × 75 μm inner diameter column packed with Acclaim PepMap RSLC C18 resin (Thermo Scientific, USA). Mobile phases were 0.1% v/v formic acid in water (eluent A) and 0.1% v/v formic acid in acetonitrile/water 4/1 v/v (eluent B), running at a total flow rate of 300 nL/min. A linear gradient started 20 min after sample loading; eluent B ramped from 3% to 40% v/v over 40 min, and from 40% to 80% v/v over 5 min. Mass spectra were acquired in the range *m/z* 400–2,000. Peptides were fragmented by collision-induced dissociation and subjected to data-dependent product ion scanning, allowing dynamic exclusion (repeat count 1 and exclusion duration 60 s) over the three most abundant ions. Mass isolation window and collision energy were set to *m/z* 3 and 35%, respectively.

Raw mass data were searched by means of MASCOT search engine (version 2.2, Matrix Science, UK) within the Proteome Discoverer software package (Thermo Scientific, USA) against the NCBI protein sequence database of all organisms belonging to the *Coleoptera* order, plus the UniProtKB entries from *T*. *molitor* and most common protein contaminants. Database searches were performed by using carbamidomethylation of cysteine as fixed protein modification and oxidation of methionine as variable modification, a mass tolerance value of 1.8 Da for precursor ions and of 0.8 Da for MS/MS fragments, trypsin as proteolytic enzyme, and a missed cleavage maximum value of 2. All other parameters were left as default. At least two sequenced peptides with an individual peptide expectation value less than 0.05, which corresponds to a confidence level for peptide attribution greater than 95%, determined the identification of protein candidates. In all cases, spectra visualization with manual verification of fragmentation attribution was performed to assign the protein candidates [23].

## Results and discussion

### Color formation of insect extracts

**Fig 2:**
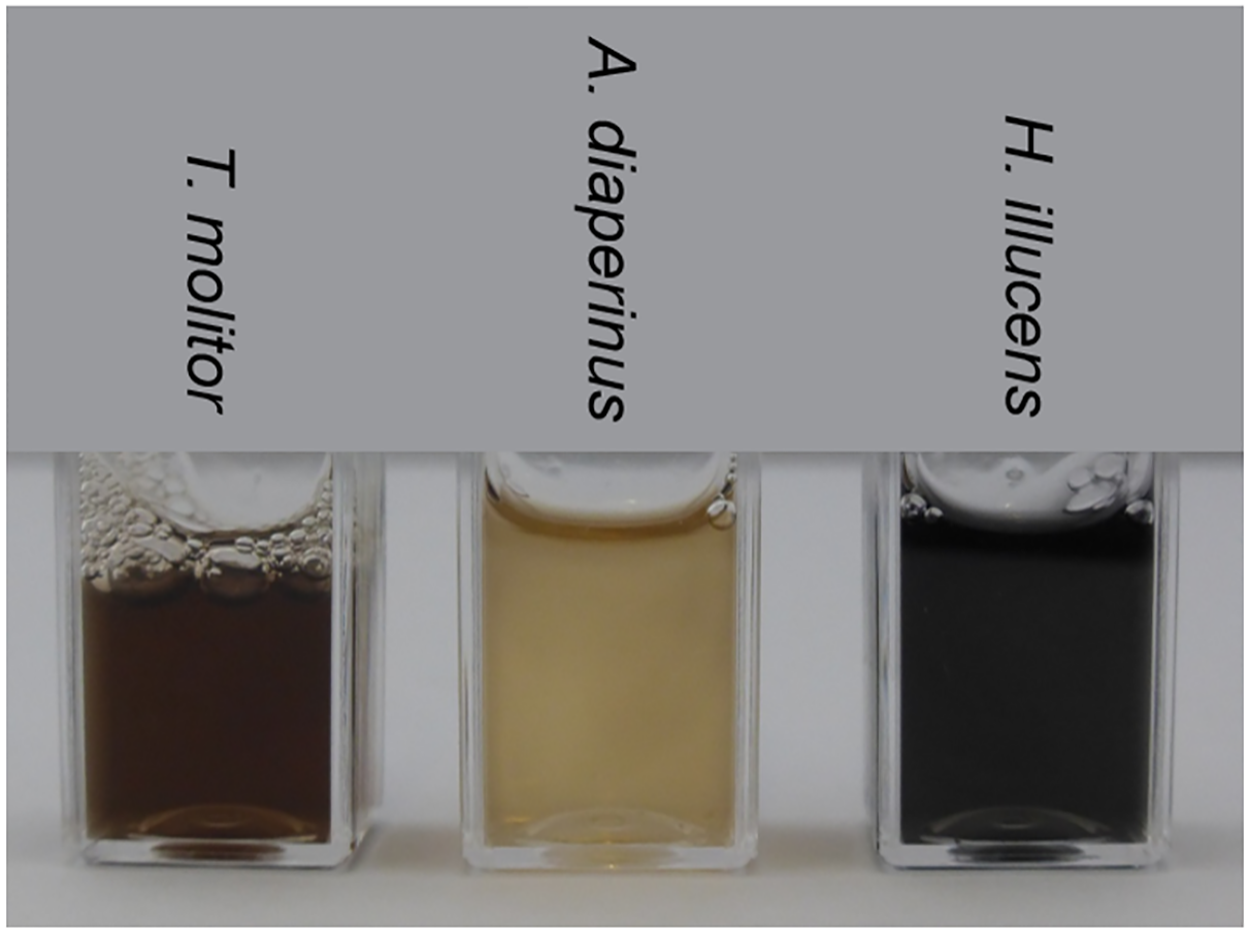
Color formation directly after grinding in MilliQ water and centrifugation of *T*. *molitor*, *A*. *diaperinus* and *H*. *illucens*.

Color formation upon grinding insects has been shown before for different insects species [4]. In Fig 2, the color of the three insects species used in this research is shown directly after grinding in MilliQ and centrifugation. *A*. *diaperinus* had the lightest color, whereas *T*. *molitor* showed a darker brown color and *H*. *illucens* was the darkest, with an almost black appearance.

### Optimal pH value for enzymatic reactions

**Fig 3:**
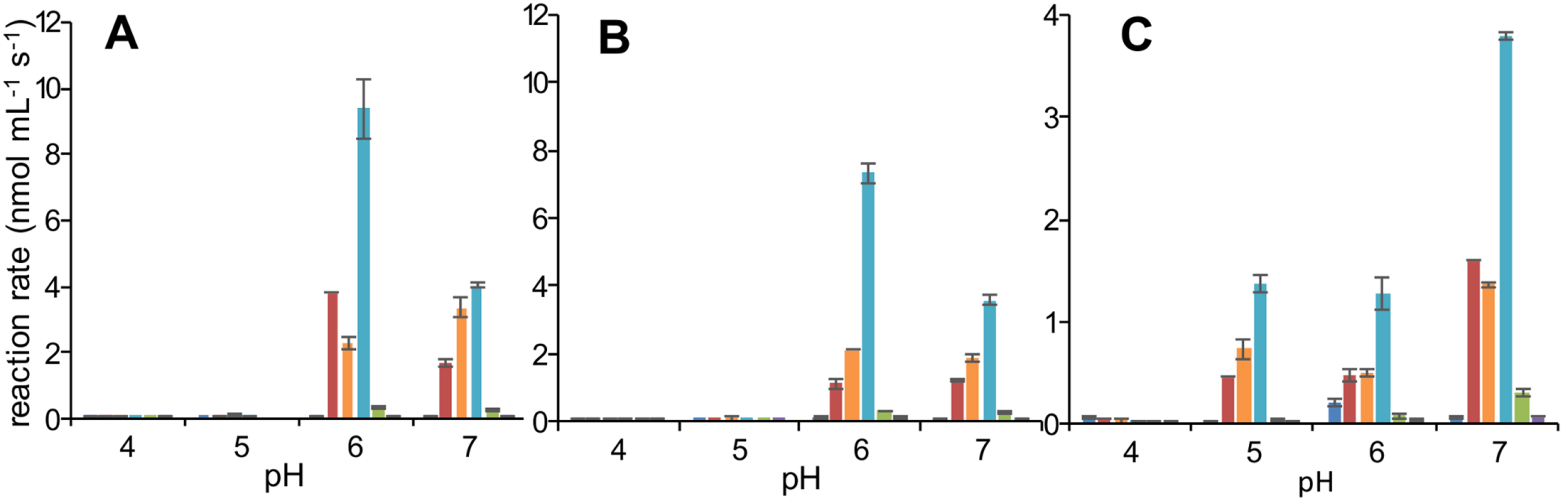
Effect of pH on specific oxidative enzyme activity (nmol mL^-1^s^-1^) of enzyme extracts from the larvae of *T*. *molitor* (A), *A*. *diaperinus* (B) and *H*. *illucens* (C). L-DOPA (red), L-DOPA+H_2_O_2_ (orange), L-dopamine (light blue), L-tyrosine (green) or ABTS (purple) were used as substrates and buffer (dark blue) as negative control (n = 2, error bars represent absolute deviation).

Different phenolic substrates were used to get an indication of the type of enzymes contributing to browning in *T*. *molitor*, *A*. *diaperinus* or *H*. *illucens*, as shown in Table 1. In insects, most phenolic compounds are derived from L-tyrosine and are often modified by enzymes and/or coupled with polar substituents to increase solubility, such as phosphate, glucose or β-alanine [24]. Here, the corresponding unconjugated phenolic structures were used for specific enzyme activity determinations. In particular, each enzyme activity was determined in the pH range of 4–7 for each of the substrates (Fig 3). Activities above pH 7 were not considered, as auto-oxidation of phenolic compounds might occur. Enzyme activities were assayed based on oxygen consumption during the reaction. In general, the highest enzyme activities were observed in the pH range 6–7.

*T*. *molitor* showed a higher oxidative enzyme activity towards L-dopamine of 9.4 nmol mL^-1^s^-1^ compared to 7.3 nmol mL^-1^s^-1^ for *A*. *diaperinus*, whereas *H*. *illucens* had lowest oxidative enzyme activity towards L-dopamine of 3.8 nmol mL^-1^s^-1^. Despite having the lowest enzyme activity, *H*. *illucens* showed the darkest color as shown in Fig 2. This clearly suggests that the color in this species is not only due to enzymatic browning, and that other factors should also be considered.

No enzymatic browning activities were observed at pH 4 for all three species. Only *H*. *illucens* showed activity at pH 5 and had the highest activity at pH 7, whereas *T*. *molitor* showed the highest activity at pH 6. For *A*. *diaperinus*, the highest activity was found at pH 6 for L-dopamine, whereas no significant difference was found for L-DOPA between pH6 and 7. The highest enzyme activities were found around the physiological pH of the larvae, which were 6.5, 6.4 and 7.0 for *T*. *molitor*, *A*. *diaperinus* and *H*. *illucens*, respectively. As the activation of browning enzymes might be related to an immune response in insects, a peak of activities around this pH seems to serve the animal physiology.

Regarding substrate specificity, the highest activity was observed on the diphenol L-dopamine, which was followed by L-DOPA, as shown before [16]. Addition of H_2_O_2_ to L-DOPA substrate showed comparable activity as without at the various pH values; this indicated that H_2_O_2_ is not necessary for the enzyme reaction, which excluded peroxidase activity as a main contributor to enzymatic browning. Low activity was found on the monophenol L-tyrosine. The hydroxylation reaction is usually lower compared to oxidation of the *ortho*-diphenol, due to the occurrence of a lag phase [17]. This is generally caused by the redox state of the copper in the enzyme’s active site. Only the *oxy*-state is able to hydroxylate a monophenol into a diphenol, whereas a diphenol can be oxidized in both *meth*- and *oxy*-state [25]. Activities on both mono- and diphenols were indicative for the presence of phenoloxidase. No activity was observed on ABTS, which indicated the lack of laccases in the insect extracts. The lack of activity assayed in the buffer without substrate showed that the oxygen consumption is correlated with the substrate added and was not due to the presence of endogenous phenolic compounds.

### Fractionation of enzymatic activities

**Fig 4:**
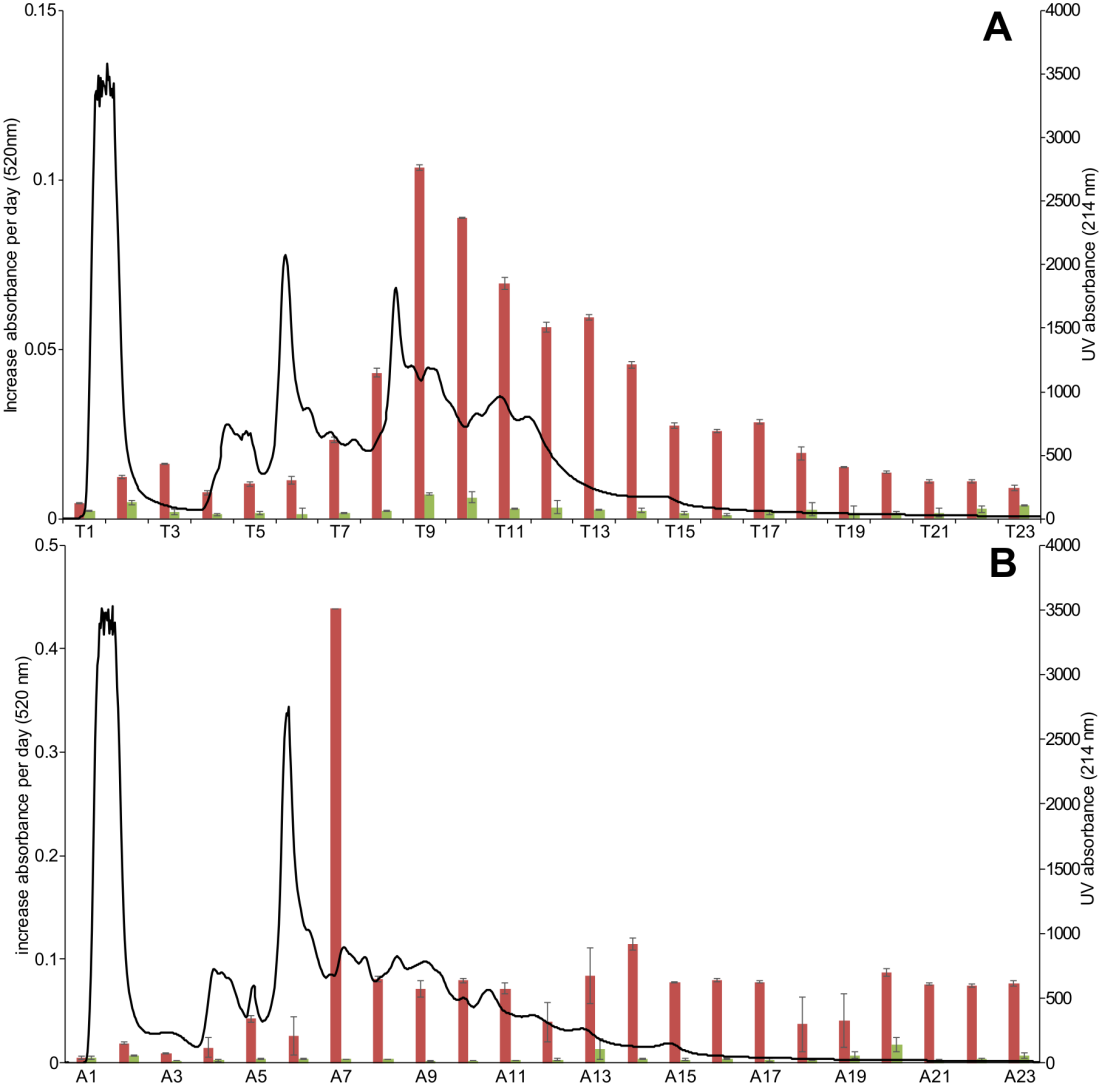
AEC fractionation pattern (black line) at 214 nm for *T*. *molitor* (A) and *A*. *diaperinus* (B). The panels also report the increase of absorbance at 520 nm per day for each fraction, when assayed with L-DOPA (red) and L-tyrosine (green) (n = 2), error bars represent absolute deviation).

Based on oxygen consumption measurements, the activity on mono- and diphenols coincided, independent of pH (Fig 3). This observation indicated the presence of a phenoloxidase alone, or of multiple enzymes working in synergy to convert the above mentioned substrates. Therefore, enzyme extracts were prepared and fractionated at pH values corresponding to the highest activity with the aim to unveil possible enzymes not separated in measurements performed with crude protein extracts. Thus, enzyme extracts of *T*. *molitor* and *A*. *diaperinus* were prepared at pH 6, whereas *H*. *illucens* was prepared at pH 7. The fractionation profiles at 214 nm for *T*. *molitor* and *A*. *diaperinus* are shown in Fig 4. The enzymatic activities in *H*. *illucens* were very low and no pattern was observed (data not shown). Chromatographic fractions were collected and incubated with L-tyrosine and L-DOPA to check for coinciding activities. Each fraction with high activity towards L-DOPA also showed a (relatively) low activity towards monophenolic L-tyrosine. This indicated that the fractions contained one enzyme, phenoloxidase, with both diphenolase and monophenolase activity. Therefore, it seems unlikely that a separate L-tyrosine hydroxylase and diphenolase (or catecholase) is responsible for the enzymatic browning of *T*. *molitor* and *A*. *diaperinus*.

### Determination of L-DOPA, L-dopamine and dopachrome formation

As reaction products L-DOPA and L-dopamine are colorless and cannot be revealed by spectrophotometry, the potential occurrence of tyrosine hydroxylase and DOPA-decarboxylase activities in *T*. *molitor* extracts was assayed qualitatively by UHPLC-MS analysis. The pooled fractions were incubated with L-tyrosine and L-DOPA. None of the incubations with L-DOPA resulted in the formation of L-dopamine, thus excluding the occurrence of DOPA decarboxylase activity therein. Tyrosine hydroxylase was also not found, as both L-DOPA and dopachrome were formed upon incubation of L-tyrosine pool T_III_. Tyrosine hydroxylase was previously described in *T*. *castaneum* as pterin-dependent species [20]. The absence of this enzyme activity in *T*. *molitor* might be due to the absence of tetrahydrobiopterin cofactor during enzyme extract preparation phases [26].

Furthermore, the presence of phenoloxidase activity was confirmed in pool T_III_ for *T*. *molitor*, as both L-DOPA and dopachrome was formed as a result of hydroxylation of L-tyrosine and further oxidation of L-DOPA, respectively. Phenoloxidase was not confirmed in the other fractions as only activity on L-DOPA and not on L-tyrosine was found for pool T_IV_ and only minor activity on L-DOPA was observed with pool T_v,_

### Presence of L-DOPA-active enzyme bands using native PAGE

**Fig 5:**
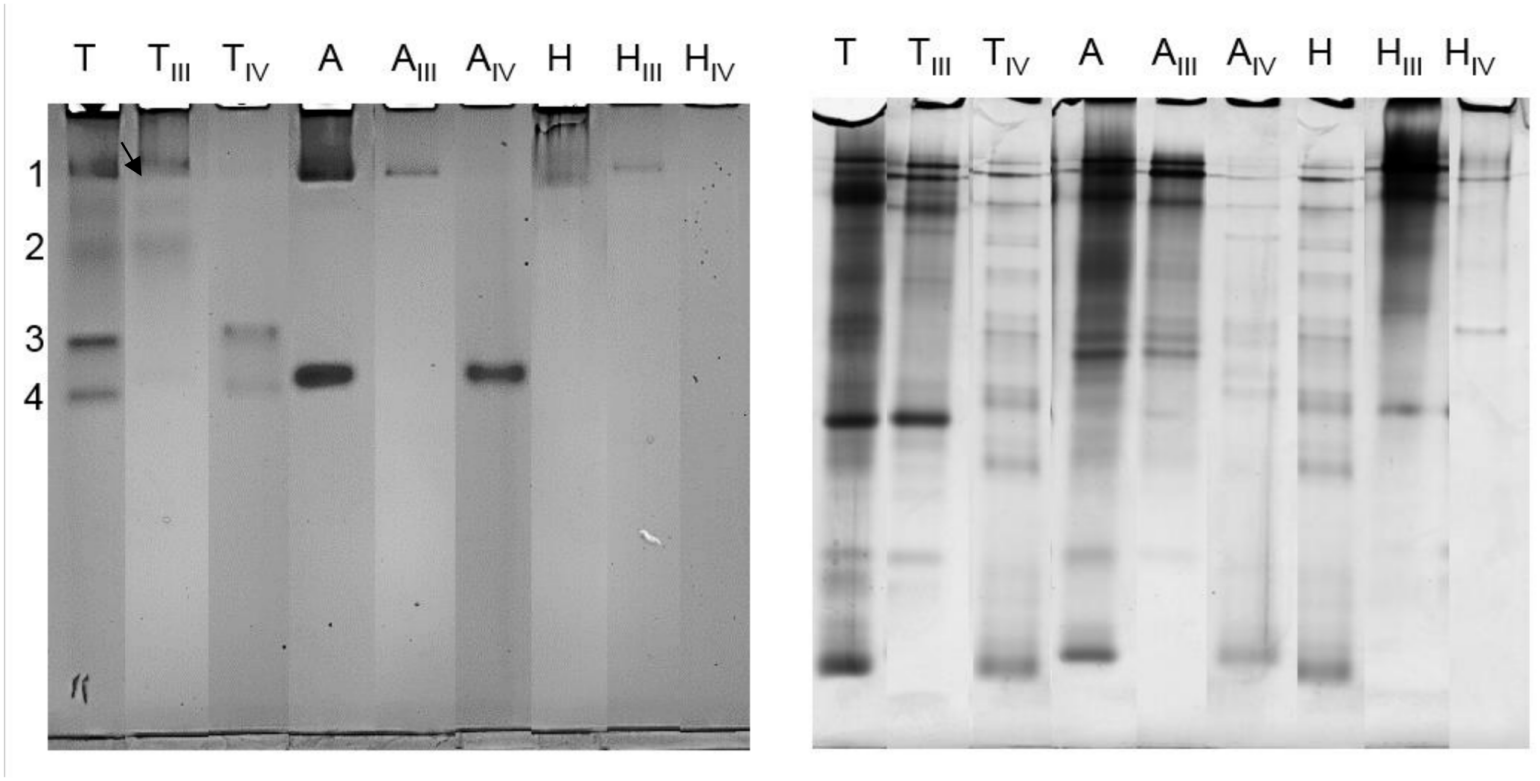
Native PAGE stained with 3 mM L-DOPA (left) showed active bands for extract of *Tenebrio molitor* (T), *Alphitobius diaperinus* (A) and *Hermetia illucens* (H). Two pooled active fractions from the same insects were subjected to PAGE analysis (T_III_, T_IV_, A_III_, A_IV_, H_III_ and H_IV_). Numbering on the left highlights the bands excised and further subjected to proteomic analysis. A similar gel was stained with Coomassie (right).

To confirm the presence of phenoloxidase activity, extracts from *T*. *molitor*, *A*. *diaperinus* and *H*. *illucens*, and corresponding pooled fractions active on L-tyrosine and L-DOPA (Table 2), were also subjected to native PAGE, and the bands were tested for positive staining with L-DOPA. *T*. *molitor* samples showed the occurrence of 4 evident active bands (Fig 5), whereas *A*. *diaperinus* counterparts showed 2 active bands. In *H*. *illucens* samples, no significant enzymatic activity was observed. Parallel native PAGE experiments followed by staining with L-tyrosine and ABTS did not show positive bands (data not shown). Pooled AEC fractions showed separation of the active bands. Thus, AEC fractionation separated a number of browning-related enzyme activities. Besides, it removed some protein contaminants, which is clearly shown upon staining of the native page gel with coommassie (Fig 5). Extra identification was done by inhibition of phenoloxidases [27]. Besides extracts with corresponding active phenoloxidase, an extract was prepared in presence of a well-known phenoloxidase, namely sodium bisulfite. Sulfite is known to irreversibly inhibit the enzyme by binding in the active site of phenoloxidase [28]. Native PAGE of these extracts did not show any active bands as shown in supporting information S1 Fig, contrary to the extract without sodium bisulfite, confirming inhibition of insect phenoloxidase by sodium bisulfite.

**Table 2:** Reaction products formed after incubation of pooled fractions T_I-V_ from *T*. *molitor* with substrates L-tyrosine and L-DOPA; + formed, × not found, = substrate constant, − substrates decreases, n/a not applicable.

Sample *Tenebrio molitor*	L-tyrosine	L-DOPA	Dopachrome	L-dopamine
T_I_ + L-DOPA	n/a	=	×	×
T_I_ + L-tyrosine	=	×	×	×
T_II_ + L-DOPA	n/a	=	×	×
T_II_ + L-tyrosine	=	×	×	×
T_III_ + L-DOPA	n/a	*−*	+	×
T_III_ + L-tyrosine	*−*	+	+	×
T_IV_ + L-DOPA	n/a	*−*	+	×
T_IV_ + L-tyrosine	=	×	+	×
T_V_ + L-DOPA	n/a	=	×	×
T_V_ + L-tyrosine	=	*−*	+	×

Due to the occurrence of reduced levels of protein contaminants as shown with Coomassie staining (Fig 5) in *T*. *molitor*, active PAGE bands obtained from the lanes with the pooled fractions were excised from the gel and further subjected to proteomic analysis for enzyme identification. It should be mentioned that poor and incomplete genetic data are available for the species under investigation, including enzymes involved in tyrosine metabolism. Nevertheless, proteomic analysis confirmed the occurrence in band 1 indicated with an arrow (Fig 5) of *T*. *molitor* phenoloxidase (also called tyrosinase) formed from pro-phenoloxidase (Table 3 and S1 Table), as the inactive form was only present in the database. The phenoloxidase form was likely activated by cleaving off a 3 kDa peptide from the enzyme by a serine protease, as the native page showed a colored band [29]. This enzyme contains six histidine residues responsible for the copper binding in its active site. This active site is similar to another well studied phenoloxidase from *Bombyx mori* (silk moth), even though this species is from a different order. These phenoloxidase share a sequence identity of 58%. Band 1 also contained two other proteins related to browning processes, namely *T*. *molitor* early-staged encapsulation inducing protein and melanization-related protein (Table 3 and S1 Table). The first component was already described as a phenoloxidase activator that is involved in defense responses [30]. The second one is a vitellogenin-like protein that was demonstrated to enhance melanin biosynthesis [12]; it seems to react with oxidized phenolics generated as result of phenoloxidase action, but the mechanistic details underlying this process are still unclear. It is worth mentioning that three browning-related enzymes mentioned above occurred within the same native PAGE band; further studies are necessary to determine if this finding was a coincidence or was due to the occurrence of a stable protein complex of these proteins in band 1.

**Table 3:** Browning related proteins identified in *T*. *molitor* bands from native PAGE (Fig 4).

	Band 1			Band 2
*NCBI or UniProtKB accession code*	Q9Y1W5	Q9NDN7	L7US91	Q9NL84
*T*. *molitor* protein	86 kDa early-staged encapsulation inducing protein	Melanization-related protein	Pro-phenoloxidase	Dopa decarboxylase
**Amino acids**	754	1439	684	475
**Theoretical mass [kDa]**	90.6	167.7	79.1	53.5
**Theoretical pI**	7.09	6.86	8.25	6.15
**Sequence coverage (%)**	14.99	3.68	5.99	12.63
**Unique Peptides**	9	4	3	4
**Mascot score**	223.41	114.59	124.81	98.65

Proteomic analysis also ascertained the occurrence of *T*. *molitor* DOPA decarboxylase in band 2 (Fig 5) (Table 3 and S1 Table). This enzyme was previously reported in *T*. *molitor* [21]; however, its enzyme activity was detected only after bacterial infection in the larvae and when the cofactor pyridoxal phosphate was added to the enzyme incubation [21], contrarily to what was done in this study. As this cofactor is necessary for activation, DOPA decarboxylase is not responsible for the browning during protein extraction and it cannot be responsible for band staining. Moreover, it was shown by UHPLC-MS that dopachrome and no L-dopamine was formed during incubation with L-DOPA. In addition to this, the bands on native page were also formed with L-dopamine as substrates, confirming the oxidative activity towards diphenolic substrates. No proteins related to enzymatic browning were identified in bands 3 and 4 (S1 Table). This might be due to the above-mentioned lack of complete information on *T*. *molitor* genome.

This research aimed at investigating the relevant enzymes that cause browning during the grinding of insects using different specific substrates. Identification of the responsible enzyme is a prerequisite to develop targeted strategies to inhibit undesired browning. All together our results indicated that phenoloxidase is an important enzyme in causing browning during grinding of *Tenebrio molitor* and most likely *Alphitobius diaperinus*.

## Supporting information

S1 Fig Native PAGE stained with 3 mM L-DOPA (left) showed no active bands for extracts treated with sodium bisulfite from *Tenebrio molitor* (T_s_), *Alphitobius diaperinus* (A_s_) and *Hermetia illucens* (H_s_). A similar gel was stained with Coomassie (right).
(DOCX)

S1 Table Results of proteomic analyses performed on native PAGE bands from *T*. *molitor* showing positive staining with L-DOPA. The corresponding NCBI or UniProtKB accession code, protein name, number of amino acids, theoretical molecular mass and pI value, sequence coverage (%), detected unique peptides and Mascot score are reported.
(DOCX)
