# Correction: Involvement of phenoloxidase in browning during grinding of *Tenebrio molitor* larvae

**DOI:** 10.1371/journal.pone.0192015

**Published:** 2018-01-25

**Authors:** Renske H. Janssen, Catriona M. M. Lakemond, Vincenzo Fogliano, Giovanni Renzone, Andrea Scaloni, Jean-Paul Vincken

S1 Fig is omitted from the Supporting Information files. It can be viewed below.

## Supporting information

S1 Fig Native PAGE stained with 3 mM L-DOPA (left) showed no active bands for extracts treated with sodium bisulfite from *Tenebrio molitor* (T_s_), *Alphitobius diaperinus* (A_s_) and *Hermetia illucens* (H_s_). A similar gel was stained with Coomassie (right).
(TIF)
