# Supplementary material for: Involvement of phenoloxidase in browning during grinding of Tenebrio molitor larvae
Source: PLoS One. 2017 Dec 15;12(12):e0189685. doi: 10.1371/journal.pone.0189685 (PMC5731683; doi:10.1371/journal.pone.0189685)
Supplement: S1 Fig — A similar gel was stained with Coomassie (right). (DOCX) [file pone.0189685.s001.docx]

# Supporting information: Involvement of phenoloxidase in browning during grinding of *Tenebrio molitor* larvae

Renske H. Janssen, Catriona M.M. Lakemond, Vincenzo Fogliano, Giovanni Renzone, Andrea Scaloni, Jean-Paul Vincken

**S1 Fig. Native PAGE stained with 3 mM L-DOPA (left) showed no active bands for extracts treated with sodium bisulfite from *Tenebrio molitor* (T_s_), *Alphitobius diaperinus* (A_s_) and *Hermetia illucens* (H_s_**). **A similar gel was stained with Coomassie (right).**
