# Supplementary material for: Involvement of phenoloxidase in browning during grinding of Tenebrio molitor larvae
Source: PLoS One. 2017 Dec 15;12(12):e0189685. doi: 10.1371/journal.pone.0189685 (PMC5731683; doi:10.1371/journal.pone.0189685)
Supplement: S1 Table — The corresponding NCBI or UniProtKB accession code, protein name, number of amino acids, theoretical molecular mass and pI value, sequence coverage (%), detected unique peptides and Mascot score are reported. (DOCX) [file pone.0189685.s002.docx]

# Supporting information: Involvement of phenoloxidase in browning during grinding of *Tenebrio molitor* larvae

Renske H. Janssen, Catriona M.M. Lakemond, Vincenzo Fogliano, Giovanni Renzone, Andrea Scaloni, Jean-Paul Vincken

**S1 Table. Results of proteomic analyses performed on native PAGE bands from *T. molitor* showing positive staining with L-DOPA. The corresponding NCBI or UniProtKB accession code, protein name, number of amino acids, theoretical molecular mass and pI value, sequence coverage (%), detected unique peptides and Mascot score are reported.**

| Accession | Description | # AAs | Theor. Mass (kDa) | Theor. pI | Coverage (%) | # Unique Peptides | # PSMs | Score |
| --- | --- | --- | --- | --- | --- | --- | --- | --- |
| Band 1 |  |  |  |  |  |  |  |  |
| 642931317 | Myosin heavy chain, muscle isoform X28 [*Tribolium castaneum*] | 1948 | 222.3 | 6.09 | 10.93 | 16 | 33 | 377.36 |
| Q9Y1W5 | 86 kDa early-staged encapsulation inducing protein OS=*Tenebrio molitor* GN=enp-6 | 754 | 90.6 | 7.09 | 14.99 | 9 | 22 | 223.41 |
| 91082465 | 60 kDa heat shock protein, mitochondrial [Tribolium castaneum] | 574 | 61.1 | 5.55 | 19.86 | 7 | 16 | 308.35 |
| Q95PI7 | Hexamerin 2 OS=*Tenebrio molitor* | 702 | 84.5 | 6.68 | 7.41 | 4 | 9 | 119.22 |
| 642919317 | Alpha-actinin, sarcomeric isoform X1 [Tribolium castaneum] | 897 | 103.7 | 5.87 | 7.47 | 5 | 9 | 146.56 |
| 642926018 | ATP-citrate synthase isoform X2 [*Tribolium castaneum*] | 1089 | 119.5 | 7.28 | 5.97 | 4 | 8 | 92.19 |
| 550249776 | Heat shock protein, mitochondrial [*Anoplophora glabripennis*] | 366 | 39.4 | 7.20 | 15.30 | 3 | 8 | 165.88 |
| Q9NDN7 | Melanization-related protein OS=*Tenebrio molitor* GN=160 kDa MRP [Q9NDN7_TENMO] | 1439 | 167.7 | 6.86 | 3.68 | 4 | 6 | 114.59 |
| 237681135 | Catalase-like [*Tribolium castaneum*] | 501 | 56.1 | 8.19 | 8.98 | 3 | 4 | 84.59 |
| A0A0B5IPQ8 | C1 family cathepsin B33 OS=*Tenebrio molitor* | 335 | 36.4 | 5.01 | 8.66 | 2 | 4 | 100.56 |
| 642926112 | Apolipophorins [*Tribolium castaneum*] | 3334 | 370.2 | 8.31 | 0.69 | 2 | 3 | 73.83 |
| 5902775 | Alpha-amylase [*Tenebrio molitor*] | 471 | 51.2 | 4.74 | 4.25 | 2 | 3 | 64.23 |
| 91088023 | Glyceraldehyde-3-phosphate dehydrogenase 2 [*Tribolium castaneum*] | 334 | 35.4 | 8.25 | 9.58 | 2 | 3 | 76.89 |
| 189237685 | Proteasome subunit alpha type-7-1 [*Tribolium castaneum*] | 249 | 28.1 | 8.18 | 11.65 | 2 | 3 | 96.15 |
| 550249068 | Glutamate dehydrogenase, mitochondrial [*Anoplophora glabripennis*] | 548 | 60.9 | 8.53 | 5.47 | 2 | 3 | 86.35 |
| L7US91 | Prophenoloxidase OS=*Tenebrio molitor* GN=PPO | 684 | 79.1 | 8.25 | 5.99 | 3 | 3 | 124.81 |
| 546686306 | Hypothetical protein D910_00056 [*Dendroctonus ponderosae*] | 960 | 103.7 | 5.30 | 3.13 | 3 | 3 | 122.47 |
| 546680166 | Hypothetical protein D910_07850 [*Dendroctonus ponderosae*] | 365 | 40.6 | 7.99 | 8.22 | 2 | 2 | 83.61 |
| 546679426 | Hypothetical protein D910_07249 [*Dendroctonus ponderosae*] | 259 | 28.8 | 5.82 | 8.88 | 2 | 2 | 56.84 |
| Q8I6J9 | Masquerade-like serine proteinase homologue OS=*Tenebrio molitor* | 444 | 48.8 | 6.30 | 5.86 | 2 | 2 | 50.78 |
| Band 2 |  |  |  |  |  |  |  |  |
| 550249393 | Heat shock 70 protein cognate 3 [*Anoplophora glabripennis*] | 657 | 72.8 | 5.19 | 15.98 | 8 | 17 | 158.61 |
| 5902775 | Alpha-amylase [*Tenebrio molitor*] | 471 | 51.2 | 4.74 | 15.07 | 5 | 10 | 169.18 |
| Q9NL84 | Dopa decarboxylase OS=*Tenebrio molitor* GN=dopa decarboxylase | 475 | 53.5 | 6.15 | 12.63 | 4 | 7 | 98.65 |
| 642937729 | Tropomyosin-2 isoform X15 [*Tribolium castaneum*] | 283 | 32.7 | 4.73 | 12.72 | 3 | 6 | 93.09 |
| Q27013 | 28 kDa desiccation stress protein OS=*Tenebrio molitor* [Q27013_TENMO] | 225 | 24.8 | 5.53 | 20.89 | 5 | 6 | 104.98 |
| 91080775 | Rab GDP dissociation inhibitor alpha [*Tribolium castaneum*] | 443 | 49.9 | 5.60 | 5.19 | 2 | 5 | 77.65 |
| 724090709 | Tropomyosin-1, partial [*Monochamus alternatus*] | 256 | 29.1 | 4.93 | 11.33 | 2 | 5 | 85.48 |
| Q8MPF2 | Triosephosphate isomerase OS=*Tenebrio molitor* GN=tpi | 247 | 26.7 | 6.35 | 10.12 | 2 | 5 | 57.01 |
| 625295138 | Heat shock cognate protein 70, partial [*Propylea japonica*] | 209 | 22.7 | 5.33 | 19.14 | 3 | 4 | 69.34 |
| 478257952 | Hypothetical protein YQE_05244, partial [*Dendroctonus ponderosae*] | 432 | 48.6 | 4.78 | 7.41 | 2 | 4 | 145.15 |
| 642923291 | Uncharacterized protein C05D11.1-like [*Tribolium castaneum*] | 1022 | 115.9 | 5.50 | 2.15 | 2 | 4 | 68.37 |
| 219873007 | Heat shock protein 90 [*Harmonia axyridis*] | 717 | 82.2 | 5.02 | 5.72 | 3 | 4 | 78.38 |
| 270002786 | Alpha spectrin [*Tribolium castaneum*] | 2415 | 278.5 | 5.20 | 1.49 | 3 | 4 | 79.00 |
| 730042794 | Histone 4, partial [*Sternopriscus wallumphilia*] | 48 | 5.6 | 9.60 | 62.50 | 3 | 4 | 79.51 |
| 381414109 | Actin, partial [*Merizodus soledadinus*] | 339 | 37.6 | 5.36 | 12.09 | 3 | 3 | 89.58 |
| 157102538 | Histone H3, partial [*Deronectes aubei aubei*] | 102 | 11.4 | 10.43 | 13.73 | 3 | 3 | 59.72 |
| 91088023 | Glyceraldehyde-3-phosphate dehydrogenase 2 [*Tribolium castaneum*] | 334 | 35.4 | 8.25 | 9.58 | 2 | 3 | 93.58 |
| C5H0E3 | Prolyl carboxypeptidase OS=*Tenebrio molitor* | 488 | 55.1 | 5.05 | 5.53 | 2 | 2 | 61.83 |
| Band 3 |  |  |  |  |  |  |  |  |
| 5902775 | Alpha-amylase [*Tenebrio molitor*] | 471 | 51.2 | 4.74 | 4.67 | 2 | 4 | 58.62 |
| 91089297 | Membrane-bound alkaline phosphatase [*Tribolium castaneum*] | 503 | 55.1 | 5.58 | 5.37 | 2 | 4 | 82.96 |
| Q27013 | 28 kDa desiccation stress protein OS=*Tenebrio molitor* | 225 | 24.8 | 5.53 | 15.11 | 3 | 3 | 74.60 |
| Band 4 |  |  |  |  |  |  |  |  |
| 5902775 | Alpha-amylase [*Tenebrio molitor*] | 471 | 51.2 | 4.74 | 8.92 | 4 | 7 | 73.99 |
| Q7YZB9 | Chitinase OS=*Tenebrio molitor* | 367 | 39.5 | 4.55 | 7.90 | 2 | 3 | 83.50 |
| Q9GSE6 | Beta-glucosidase (Fragment) OS=*Tenebrio molitor* | 502 | 57.7 | 4.59 | 4.58 | 2 | 3 | 71.25 |
