# Supplementary figures and images for: Correction: Involvement of phenoloxidase in browning during grinding of Tenebrio molitor larvae
Source: PLoS One. 2018 Jan 25;13(1):e0192015. doi: 10.1371/journal.pone.0192015 (PMC5785002; doi:10.1371/journal.pone.0192015)

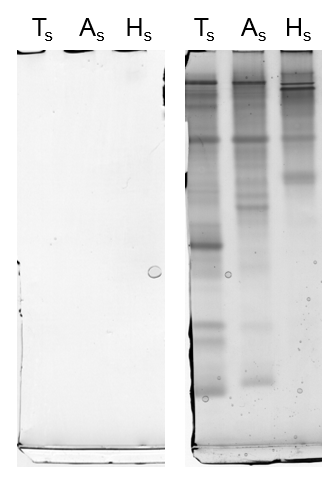

Supplement: S1 Fig — Native PAGE stained with 3 mM L-DOPA (left) showed no active bands for extracts treated with sodium bisulfite from Tenebrio molitor (Ts), Alphitobius diaperinus (As) and Hermetia illucens (Hs). A similar gel was stained with Coomassie (right). (TIF) [file pone.0192015.s001.tif]
